# Trajectories of Short Physical Performance Battery Are Strongly Associated with Future Major Mobility Disability: Results from the LIFE Study

**DOI:** 10.3390/jcm9082332

**Published:** 2020-07-22

**Authors:** Joshua D. Brown, Wei-Hsuan Lo-Ciganic, Hui Shao, Marco Pahor, Todd M. Manini

**Affiliations:** 1 Center for Drug Evaluation & Safety, Department of Pharmaceutical Outcomes & Policy, University of Florida College of Pharmacy, Gainesville, FL 32610, USA; wlociganic@cop.ufl.edu (W.-H.L.-C.); hui.shao@cop.ufl.edu (H.S.); 2 Institute on Aging, Department of Aging and Geriatric Research, University of Florida College of Medicine, Gainesville, FL 32610, USA; mpahor@ufl.edu (M.P.); tmanini@ufl.edu (T.M.M.)

**Keywords:** group-based trajectory models, major mobility disability, Short Physical Performance Battery, LIFE Study, physical activity interventions

## Abstract

Short Physical Performance Battery (SPPB) assessment is a widely used measure of lower extremity function, strength, and balance. In the Lifestyles Interventions and Independence for Elders (LIFE) Study, baseline SPPB and changes throughout the trial were strongly associated with major mobility disability (MMD). This study further investigated this association by identifying trajectories of SPPB and evaluating the predictive validity of SPPB trajectories for future MMD. Participants (*n* = 1635) aged 70–89 years were randomized to a physical activity or health education intervention and assessed every 6 months for MMD. We used group-based trajectory models (GBTMs) to identify trajectories of a binary outcome for a decrease from baseline SPPB of ≥1. Multinomial logistic regression explored baseline factors associated with group membership. Survival analyses evaluated the association between trajectories with MMD. The GBTM identified a 3-group model which included a “No Decline” group (46.0%), “Late Decline” group (27.7%), and an “Early Decline” group (26.3%). Adjusting for all other baseline characteristics, group assignment during the previous follow-up visit was strongly associated with MMD at the subsequent period. Comparisons between groups showed a 2-to-3-fold increase in MMD comparing the “Late” to “No” decline group and a 4-to-5-fold increase in MMD comparing the “Early” to “No” decline group. Group membership and impact on MMD was not different between intervention arms. Group-based trajectories of SPPB scores identified distinct subgroups in LIFE Study participants. Using these group assignments in outcome models were highly associated with MMD. GBTMs have potential to identify and improve prediction of aging-related decline to better design and identify patients for interventions.

## 1. Introduction

The Short Physical Performance Battery (SPPB) is a widely used assessment of lower extremity function in older adults [1]. SPPB measures balance, gait, strength, and endurance, particularly of the lower extremities by combining an eight-foot (8′) walk test, chair sitting and standing, and balance tests [1]. SPPB has been strongly associated with all-cause mortality, disability, rehospitalization and healthcare utilization in older adults making it an important assessment tool in clinical practice as it can be quickly administered and requires minimal equipment and space [2,3,4,5,6]. Since its development, it has also been widely used in observational studies and clinical trials in older adults to assess or predict aging-related and condition-specific outcomes [7].

The Lifestyle Interventions and Independence for Elders (LIFE) Study was the largest and longest randomized clinical trial to assess the impact of a physical activity intervention on mobility disability in older adults [8,9]. The study utilized SPPB measures as both inclusion criterion (SPPB ≤ 9 for inclusion) and a tertiary outcome measure taken at various points throughout follow-up. The primary outcome, major mobility disability (MMD) assessed participants’ ability to complete a 400-m walk test in 15-min without assistance. In the LIFE Study, baseline SPPB and overall changes in SPPB were important predictors of response to the physical activity intervention [10].

Group-based trajectory modeling (GBTM) is an analytic framework which posits that within a population there are a number of latent, unrecognized groups that follow a similar longitudinal pattern (trajectory) [11]. GBTM has been applied in aging research to identify, for example, risk factors for fast decline of gait speed [12], associations between long-term physical activity and mortality [13], strong associations between cognitive trajectories and healthcare burden [14], and health trajectories in older adults between countries [15]. Using GBTMs to identify distinct subgroups hold promise to provide new insights on individuals at high-risk of MMD for targeted interventions.

The purpose of this study was to expand the strong person-level associations between SPPB and MMD outcomes in the LIFE Study by identifying group trajectories of SPPB changes over time and subsequent outcomes. The study aimed to identify if groups with distinct trajectories of SPPB existed within the LIFE Study cohort, what factors predict an individual’s membership in these groups, and if group membership was associated with the primary outcome, MMD.

## 2. Methods

### 2.1. LIFE Study Overview

The LIFE Study was a multi-center, single-blind, parallel randomized trial conducted across eight centers in the United States between February 2010 and December 2013 with participant recruitment occurring between February 2010 and December 2012 [9]. Among the *n* = 1635 participants randomized during the study period, there was an average of 2.6 years of follow-up time with loss to follow-up of 4% annually [9]. The study protocol was approved by the institutional review boards of each institution. Written informed consent was obtained from all study participants. The trial was monitored by a data and safety monitoring board appointed by the National Institute on Aging. The LIFE Study was registered prior to participant enrollment in the trial (NCT01072500). Details of the study design, rationale, and characteristics of the full study population are described elsewhere [9,16]. Participants were eligible for the trial who were 70–89 years of age, scored ≤ 9 on the SPPB, were sedentary with ≤125 min of activity per week, and were able to complete the 400-m walk test within 15 min without sitting, leaning or without assistance. Reuse of the LIFE Study data for this analysis was approved under expedited review by the University of Florida Institutional Review Board (IRB201701581).

### 2.2. Intervention

Details of the study interventions were published previously [9,17]. The physical activity (PA) intervention involved walking, with a goal of 150 min per week, strength, flexibility, and balance training. The intervention included attendance at two center-based visits per week and home-based activity three to four times per week for the duration of the study. The PA sessions were individualized and progressed toward a goal of 30 min of walking daily at moderate intensity, 10 min of primarily lower-extremity strength training by means of ankle weights (2 sets of 10 repetitions), 10 min of balance training, and large muscle group flexibility exercises.

The health education (HE) control group included weekly educational workshops during the first 26 weeks, and then monthly sessions thereafter. Workshops included topics relevant to older adults, such as how to effectively negotiate the health care system, how to travel safely, preventive services and screenings recommended at different ages, where to go for reliable health information, nutrition, etc. The workshops did not include any PA topics. The program also included a 5- to 10-min instructor-led program of gentle upper extremity stretching or flexibility exercises.

### 2.3. Follow-Up Visits and Outcome Assessment

Participants were assessed for the primary outcome (MMD) every 6 months at clinic visits. Home, telephone, and proxy assessments were attempted if participants could not return to the clinic. The assessment staff were masked to the intervention assignment and remained separate from the intervention team. Participants were asked not to disclose their assigned intervention arm or talk about their interventions during the assessment.

Details of MMD ascertainment were reported previously [8]. Briefly, participants were asked to walk 400 m at their usual pace, and MMD was defined as the inability to complete the walk within 15 min without sitting and without the help of another person or walker. When MMD could not be objectively measured because of the inability of the participant to come to the clinic and absence of a suitable walking course at the participant’s home, institution, or hospital; an alternative adjudication of the outcome was based on objective inability to walk 4 m in less than 10 s, or self-, proxy-, or medical record–reported inability to walk across a room. If participants met these alternative criteria, they were considered to be unable to complete the 400-m walk within 15 min.

SPPB was measured during each clinical follow-up visit at baseline, 6, 12, 24, and 36 months. SPPB included a 400-m walking velocity, timed repeated chair stand, and three standing balance tests. Each test is assigned a score ranging from 0 to 4 (inability to complete up to best performing) and the three test scores are summed to a summary score ranging from 0 (worst performers) to 12 (best performers).

### 2.4. Group-Based Trajectory Modeling

**Figure 1:**
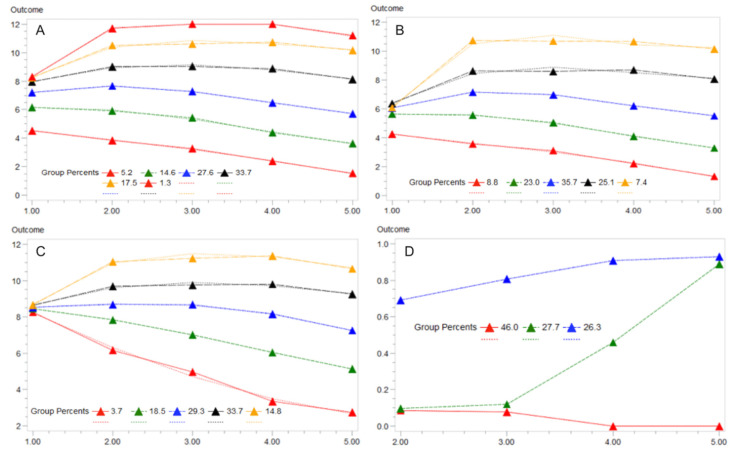
Group-based trajectory models (GBTM) of Short Physical Performance Battery (SPPB, “Outcome” on y-axis) over 5 follow-up periods in the LIFE Study (x-axis) using a censored normal distribution model. Final models (i.e., number of groups) were selected based on the best overall model fit criteria (Bayesian Information Criterion). Group membership percentages are displayed along with lines of best fit (dashed lines). (**A**) includes the full LIFE Study sample with the best model being a 6-group trajectory. (**B**,**C**) stratified the sample by SPPB ≤ 7 or > 7 given the dependence of the overall model on the initial value of SPPB. (**D**) represents the final binary failure model of the standardized outcome of ”declined SPPB” based on a reduction from baseline of ≥1 point. Note: y-axis is total SPPB score in panels A/B/C. y-axis is the probability of a ≥1-point decline in SPPB in panel D. X-axis is follow-up time in each panel.

A longitudinal file of SPPB assessment scores was organized for each individual which included the baseline, 6, 12, 24, and 36-month SPPB measurements. Given dependence of the overall group membership on baseline SPPB scores (Figure 1A), we standardized the SPPB models by using a binary indicator for whether an individual’s SPPB score decreased ≥1 point compared to baseline in each time period, which is the minimum clinically relevant difference for SPPB [7]. GBTMs with logistic function were estimated for 2–8 groups using PROC TRAJ [18] in SAS Enterprise Guide v7.1 (SAS Institute, Cary, NC, USA) which predicted the probability of having a decline in SPPB in each follow-up period. GBTM models correlate trajectories in the dependent variable (SPPB) over time to identify latent (hidden) groupings of individuals based on trajectories of this variable. No other characteristics are considered in the trajectory models. Best practices for model selection included a comparison of the Bayesian Information Criterion (BIC) between models and Nagin’s Criteria [11]. Missing observations for SPPB were treated as censored values.

Based on the overall model fit, we identified a final three group model and categorized the groups as “No Decline,” “Early Decline,” and “Late Decline” based on the visible pattern in SPPB (Figure 1D). We described baseline characteristics including intervention group (PA or HE), physical characteristics (age, sex, race (white, black, or other), body mass index (BMI, kg/m^2^), functional assessments, sleep quality (Pittsburgh Sleep Quality Index), grip strength (kg), gait speed (m/s), past medical history, and education (≥high school). Included functional assessments were previously validated measures summarized as raw scores within their individual range of values which included, for cognitive function, the Memory and Aging Telephone Screen (MATS) and the Modified Mini Mental State Examination (3MSE) and activity levels were measured via the Community Healthy Activities Model Program for Seniors (CHAMPS). Physical functioning was captured using baseline gait speed, grip strength, SPPB (score of ≤ 7), and the Pepper Assessment Tool for Disability (PAT-D). Overall current self-rated health (at baseline) was grouped based on “good, very good, or excellent” health status. Details of these assessments and their respective methodologies and score ranges can be found in the original trial deign and methods publications [8,9]. We estimated a multinomial logistic regression of these characteristics to identify characteristics associated with group membership. Adjusted odds ratios (OR) with 95% confidence intervals (CIs) are reported.

Lastly, we created a time-varying group membership variable and utilized it as the primary variable of interest in a proportional hazard regression for the MMD outcome. Group membership was identified during the prior follow-up period using the largest posterior probability of group membership. The subsequent follow-up period was assessed for the outcome (MMD). We estimated the proportional hazards model with all other baseline characteristics and reported hazard ratios (HR) and 95% CIs for the overall cohort as well as stratified by intervention group assignment.

## 3. Results

Among the 1635 LIFE participants, GBTMs of the full cohort on the raw continuous SPPB score showed the best fit with a 6-group model. However, upon visual inspection, membership in this model (and all other models estimated) was strongly dependent on the baseline SPPB score (Figure 1A). Thus, we stratified the cohort by SPPB ≤ 7 and > 7 and re-estimated these separate models (Figure 1B,C). Each model was the best fit by a 5-group model that more clearly distinguished group trajectories. Notably, the SPPB > 7 cohorts all had the same average baseline SPPB (~8) which deviated into final SPPB average values ranging from 3–11 showing clear trajectories of both increasing and decreasing SPPB values (Figure 1C).

Using a standardized measure of SPPB decline of ≥1 point, the binary models produced a 3-group model that was easily interpretable (Figure 1D). Group memberships included 46.0% of the cohort in a “No Decline” group, 27.7% in a “Late Decline” group, and 26.3% in an “Early Decline” group. The groups were overall very similar on baseline characteristics (Table 1). There were also few independent predictors of group membership in fully adjusted models (Table 2). Compared to the “No Decline” group, “Late Decline” and “Early Decline” group membership was associated with increases in age, “other” races compared to Black race, and lower SPPB scores at baseline. Self-rated health as good, very good, or excellent was associated with higher membership in the “Late Decline” group (OR = 1.94 (1.01–3.74)). There were no differences in group membership and intervention arm assignment. Increases in MATS score, grip strength, 3MSE, and female sex were positively associated with membership in the “No Decline” group while higher self-rated health, other race, and lower SPPB scores were associated with declines in SPPB groups. In the comparison between “Early” versus “Late” Decline, female sex (OR = 2.13 (1.13–4.02)) and current smoking status (OR = 3.19 (1.14–8.96)) were the only significant predictors.

**Figure 2:**
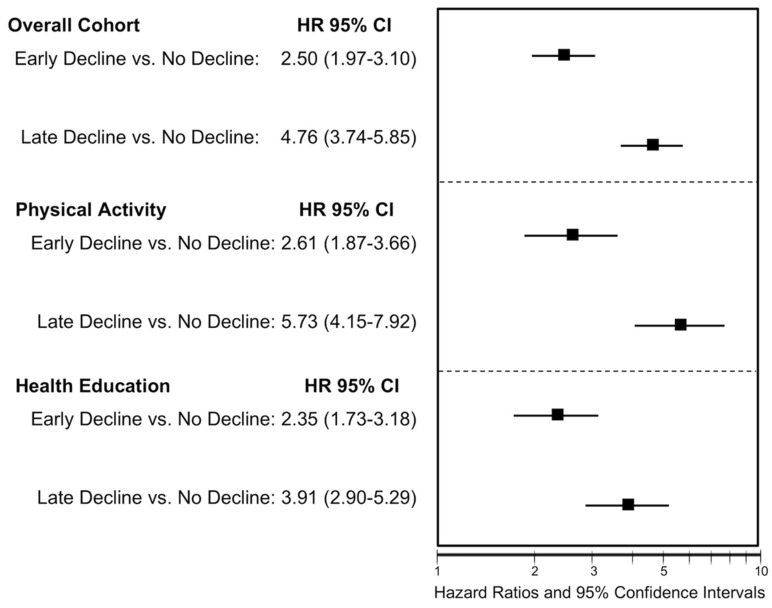
Time-varying Cox proportional hazard regression results for the association between group membership related to a SPPB decline ≥1 point with the primary outcome of major mobility disability (MMD) Note: Group membership was defined as the prior follow-up period with MMD occurring in the subsequent follow-up period. HR = Hazard ratio; CI = confidence interval. Physical Activity group was the LIFE trial intervention arm; Health Education was the control group.

Adjusting for all other baseline characteristics, prior period group assignment was strongly associated with subsequent period rates of MMD (Figure 2). Compared to the “No Decline” group, the “Late Decline group was associated with greater than two-fold increases in MMD (HR = 2.50 (1.97–3.10)) in the overall cohort and was similar when stratified intervention groups. Similarly, the “Early Decline” group was associated with 4- to 5-fold increases in the rate of MMD in the overall cohort (HR = 4.76 (3.76–5.85)) and similar in magnitude between intervention groups. This effect appeared to be greater in the PA intervention group compared to the HE controls, but the interaction term did not reach statistical significance.

## 4. Discussion

In this secondary analysis of the LIFE Study clinical trial, we identified distinct GBTMs that classify individuals into groups based on SPPB scores. These trajectories showed few factors at baseline that predicted group membership, but these groups were highly associated with the primary MMD outcome.

While the PA intervention successfully prevented MMD in the original trial, there were indicators of heterogeneity in that treatment effect [8,10]. Capturing these predictors could assist in developing better interventions or identifying higher-risk subgroups for interventions. For instance, SPPB ≤ 7 at baseline was associated with a higher risk of MMD but this group also showed a stronger response to the intervention in prior analyses [10]. Here, we utilized GBTMs as another means to explore this unique association between SPPB and MMD. While that association remained strong, we found that there were few factors related to group membership at baseline, thus, it appeared that baseline data alone could not be used to identify individuals at high risk of MMD. Additional work will evaluate simultaneous GBTMs of other physical and cognitive assessments as well as explore the impact of intervening health events (e.g., hospitalizations, fall and fractures) on GBTMs.

The results of the GBTM models as well as the regression results exploring associations with group membership confirm the observed paradoxical relationship between SPPB and the PA intervention in the LIFE Study [9]. For those with SPPB > 7 with an average baseline SPPB of approximately 8, five distinct groups were identified that could be further characterized as two groups with improvement (Figure 1C; 48.5% of those with SPPB > 7) while another 22.2% can be categorized as declining in SPPB scores over the trial period. Conversely, in those with SPPB ≤ 7 (Figure 1B), a group specifically recruited for the LIFE Study, the average baseline scores were around 6 for the five identified groups and was made up of 31.8% in groups that declined and only 32.5% characterized as improving over time.

These results are significant as it has been previously observed that the PA intervention was effective in those with SPPB ≤ 7 but not in those with higher scores [8,19]. Paired with the observation in this study that higher self-rated health was associated with belonging to the late or early decline group, a few conclusions can be made. Importantly, it is becoming apparent that in some older adults, there may be a trade-off between increased physical activity and exercise and potential harms such as injuries and fall risk [20]. These results support that these risks may correlate with baseline functioning, potentially due to the perception of ability and greater exertion leading to increased risk of injury. Rather, among those with already present limitations, approaches to exercise may be more conservative which aids in maintaining or improving baseline functioning with less risk of injury and future limitation [19]. In addition, the concept of “regression to the mean,” wherein those with lower results will tend to improve and those with higher results will tend to decline, cannot be ruled out. However, we believe this to be less likely to explain the results given the correlation of decline groups with self-rated health.

SPPB is widely used in geriatric assessments both for research and clinical purposes [1]. SPPB is an attractive physical assessment as it measures multiple components of physical strengthen and balance and is simple and quick to perform in-person or virtually with little equipment or space required. By showing that trajectories of SPPB are predictive of future MMD, we have shown that tracking an individual’s SPPB scores may be a scalable way of predicting and intervening on future issues related to mobility. We have shown, however, that aside from baseline SPPB scores, there were few predictive variables associated with how these assessments vary over time. These results are further confirmation that interventions may be most effective among those with lower baseline physical functioning and also suggests that those at higher functioning, especially those with high assessments of their own health, may be a more “risky” group for physical activity. Care should be taken in recruitment of individuals into exercise programs and education to ease into such programs may be further necessary [21]. Preventing declines in physical functioning is an important component of caring for older adults and as physical functioning is important to healthy aging and associated with healthcare outcomes, utilization and costs [22,23].

### Study Limitations

This study is strengthened by a clinically relevant sample, long follow-up, and objective outcome measurement. Limitations include assumptions made for GBTMs as well as the selection of the final trajectory models. While SPPB was measured at several intervals, differential follow-up and censoring were allowed. The model assumed censoring was random and non-informative, however, this might not always be the case. While the selection of final models was based on model fit, definitions of each group are investigator-driven which may introduce some level of subjectivity in interpretation. However, the final model represented easily interpreted group memberships. While the binary SPPB decline model standardized the measurement, it ignored improvements in SPPB which may be more predictive of future events and deserves further research. Lastly, while the analysis tracked SPPB and its association with MMD while controlling for other baseline factors, the regression does not account for other intervening health events (e.g., hospitalizations) or other time-varying characteristics. Such events would likely be on the causal pathway or follow reductions in physical functioning or be further modified by the intervention. Future research should establish the temporality between such events to understand cause and effect between incident disability and healthcare events.

## 5. Conclusions

Group-based trajectories of SPPB scores identified distinct subgroups in LIFE Study participants. The use of these group assignments in outcome models was highly predictive of the primary MMD outcome. Tracking SPPB longitudinally for an individual may provide insights into future mobility limitations and may allow identification of patients needing intervention. GBTMs have the potential to identify and improve prediction of aging-related decline to better design and identify patients for interventions.

## Figures and Tables

**Table 1:** Bivariate association of baseline characteristics with group-based trajectories for functional decline measured by a decrease of SPPB ≥ 1 in a 3-group model (Figure 1D).

Characteristics	Group Membership		
	Group 1: No Decline	Group 2: Early Decline	Group 3: Late Decline
	*n* = 752 (46.0%)	*n* = 453 (27.7%)	*n* = 430 (26.3%)
Physical activity intervention (*n* (%)]	379 (50.4%)	231 (51%)	208 (48.5%)
Age, mean (SD)	78 (5.0)	79.4 (5.2)	80.1 (5.3)
Female sex (*n* (%)]	526 (70%)	306 (67.6%)	266 (61.9%)
Race (*n* (%))			
White	543 (72.2%)	356 (78.6%)	343 (79.8%)
Black	147 (19.5%)	73 (16.1%)	66 (15.3%)
Other	62 (8.3%)	24 (5.3%)	21 (4.9%)
Education, ≥high school (*n* (%)]	492 (65.5%)	315 (69.5%)	293 (68.2%)
BMI, kg/m^2^ (mean (SD)]	30.3 (5.9)	30.5 (5.8)	29.6 (6.3)
Physical functioning assessments			
SPPB ≤ 7 ^b^ (*n* (%))	333 (44.2%)	221 (48.8%)	176 (40.9%)
Gait speed, m/s (mean (SD))	0.8 (0.2)	0.8 (0.2)	0.8 (0.2)
Grip Strength, kg (mean (SD))	24.6 (10.4)	23.1 (8.9)	22.7 (9)
CHAMPS Score (range 0–120) ^a^ (mean (SD))	16.9 (32.5)	17 (33.4)	17.4 (33.5)
PAT-D (range 1–5) ^b^ (mean (SD))	1.4 (0.4)	1.4 (0.4)	1.5 (0.4)
Cognitive functioning assessments			
3MSE (range 0–100) ^a^ (mean (SD))	92 (5.2)	91.7 (5.4)	90.8 (5.6)
PSQI (range 0–21) ^b^ (mean (SD))	5.9 (3.8)	6 (3.7)	6 (3.8)
MATS score (range 30–80) ^a^ (mean (SD))	55.1 (7.9)	52.4 (7.7)	52.2 (8)
Smoking (*n* (%))			
Never	405 (53.8%)	241 (53.1%)	218 (50.6%)
Former	320 (42.6%)	204 (45%)	198 (46.1%)
Current	27 (3.6%)	9 (1.9%)	14 (3.3%)
CVD (*n* (%))	205 (27.3%)	139 (30.8%)	146 (33.9%)
Diabetes (*n* (%))	199 (26.5%)	133 (29.3%)	126 (29.4%)
Heart attack (*n* (%))	69 (9.2%)	46 (10.1%)	42 (9.9%)
Heart failure (*n* (%))	39 (5.2%)	34 (7.5%)	23 (5.4%)
Metabolic Syndrome (*n* (%))	365 (48.5%)	242 (53.3%)	207 (48.1%)
Hypertension (*n* (%))	522 (69.5%)	321 (70.9%)	302 (70.1%)
Arthritis (*n* (%))	150 (19.9%)	75 (16.6%)	91 (21.2%)
Chronic lung disease (*n* (%))	133 (17.7%)	77 (17.1%)	71 (16.5%)
Self-Rated Health (good, very good, or excellent) (*n* (%))	462 (61.4%)	294 (65%)	296 (68.8%)

Abbreviations: SD = standard deviation; BMI = body mass index, MATS = Mobility Assessment Tool: Short Form; PAT-D = Pepper Assessment Tool for Disability; 3MSE = Modified Mini Mental Examination; PSQI = Pittsburgh Sleep Quality Index; CHAMPS = Community Healthy Activities Model Program for Seniors; CVD = cerebrovascular disease; SPPB = Short Physical Performance Battery. ^a^ Higher scores indicate higher functioning status. ^b^ Higher scores indicate lower functioning status.

**Table 2:** Multinomial logistic regression results predicting group membership based on baseline characteristics in the LIFE Study.

Characteristics	Group Membership Comparisons		
	Late Decline (Group 2) vs. No Decline (Group 1) ^1^	Early Decline (Group 3) vs. No Decline (Group 1) ^2^	Early Decline vs. Late Decline ^3^
Physical activity intervention	1.05 (0.80–1.37)	0.84 (0.64–1.11)	0.96 (0.74–1.26)
Age	1.05 (1.02–1.08) *	1.07 (1.03–1.10) *	0.98 (0.93–1.02)
Female sex	0.66 (0.44–0.98) *	0.39 (0.26–0.59) *	2.13 (1.13–4.02) *
Race			
White	0.65 (0.31–1.35)	0.9 (0.42–1.9)	1.20 (0.62–2.32)
Black	Reference	Reference	Reference
Other	1.29 (0.87–1.93)	1.63 (1.06–2.51) *	1.55 (0.51–4.71)
Education, ≥high school	1.34 (0.99–1.81)	1.29 (0.94–1.77)	0.89 (0.57–1.40)
BMI	1.01 (0.99–1.04)	0.98 (0.96–1.01)	1.00 (0.96–1.05)
MATS score	0.95 (0.93–0.98) *	0.95 (0.93–0.97) *	1.02 (0.99–1.06)
Gait speed (m/s)	1.4 (0.49–3.97)	1.22 (0.41–3.6)	2.50 (0.57–11.0)
Grip Strength	0.99 (0.97–1.01)	0.97 (0.95–0.99) *	0.99 (0.96–1.02)
PAT-D	1.1 (0.7–1.71)	1.53 (0.98–2.4)	0.87 (0.45–1.69)
3MSE	0.98 (0.95–1.00)	0.95 (0.92–0.97) *	1.03 (0.98–1.07)
PSQI	1.01 (0.97–1.04)	0.99 (0.95–1.03)	1.05 (0.99–1.11)
CHAMPS Score	1.00 (1.00–1.00)	1.00 (1.00–1.00)	1.00 (1.00–1.00)
SPPB ≤ 7	1.14 (1.04–1.25) *	1.32 (1.19–1.46) *	1.08 (0.66–1.76)
Smoking			
Never	Reference	Reference	Reference
Former	1.06 (0.8–1.4)	1.16 (0.86–1.56)	0.85 (0.55–1.32)
Current	0.44 (0.17–1.14)	1.1 (0.51–2.41)	3.19 (1.14–8.96) *
CVD	1.1 (0.79–1.55)	1.16 (0.82–1.64)	0.92 (0.55–1.52)
Diabetes	0.99 (0.71–1.39)	1.22 (0.87–1.72)	0.86 (0.53–1.39)
Heart attack	0.78 (0.45–1.33)	0.71 (0.41–1.24)	1.93 (0.92–4.08)
Heart failure	1.19 (0.63–2.23)	0.87 (0.44–1.72)	0.91 (0.34–2.46)
Metabolic Syndrome	1.11 (0.8–1.54)	1.14 (0.81–1.61)	0.98 (0.80–1.23)
Hypertension	0.95 (0.69–1.32)	1.05 (0.75–1.47)	0.90 (0.80–1.05)
Arthritis	0.69 (0.48–1.01)	1.03 (0.72–1.48)	1.12 (0.65–1.94)
Chronic lung disease	0.92 (0.64–1.32)	0.71 (0.48–1.05)	1.04 (0.60–1.81)
Self-Rated Health (>Good)	1.94 (1.01–3.74) *	1.71 (0.84–3.47)	1.13 (0.98–1.30)

Abbreviations: SD = standard deviation; BMI = body mass index, MATS = Mobility Assessment Tool: Short Form; PAT-D = Pepper Assessment Tool for Disability; 3MSE = Modified Mini Mental Examination; PSQI = Pittsburgh Sleep Quality Index; CHAMPS = Community Healthy Activities Model Program for Seniors; CVD = cerebrovascular disease; SPPB = Short Physical Performance Battery. ^1^ Odds ratios measure the likelihood of being in Group 2 vs. Group 1. ^2^ Odds ratios measure the likelihood of being in Group 3 vs. Group 1. ^3^ Odds ratios measure the likelihood of being in Group 3 vs. Group 2. * Indicates statistical significance at *p* < 0.05.

## References

[1] Guralnik J.M., Simonsick E.M., Ferrucci L., Glynn R.J., Berkman L.F., Blazer D.G., Scherr P.A., Wallace R.B. (1994). A Short Physical Performance Battery Assessing Lower Extremity Function: Association with Self-Reported Disability and Prediction of Mortality and Nursing Home Admission. J. Gerontol..

[2] Santanasto A.J., Glynn N.W., Lovato L.C., Blair S.N., Fielding R.A., Gill T.M., Guralnik J.M., Hsu F.-C., King A.C., Strotmeyer E.S. (2017). Effect of Physical Activity versus Health Education on Physical Function, Grip Strength and Mobility. J. Am. Geriatr. Soc..

[3] Bean J.F., Kiely D.K., LaRose S., Goldstein R., Frontera W.R., Leveille S. (2010). Are changes in leg power responsible for clinically meaningful improvements in mobility in older adults?. J. Am. Geriatr. Soc..

[4] Legrand D., Vaes B., Mathei C., Adriaensen W., Van Pottelbergh G., Degryse J.-M. (2014). Muscle Strength and Physical Performance as Predictors of Mortality, Hospitalization, and Disability in the Oldest Old. J. Am. Geriatr. Soc..

[5] Volpato S., Cavalieri M., Sioulis F., Guerra G., Maraldi C., Zuliani G., Fellin R., Guralnik J.M. (2010). Predictive Value of the Short Physical Performance Battery Following Hospitalization in Older Patients. J. Gerontol. Ser. A Biol. Sci. Med. Sci..

[6] Pavasini R., Guralnik J., Brown J.C., Di Bari M., Cesari M., Landi F., Vaes B., Legrand D., Verghese J., Wang C. (2016). Short Physical Performance Battery and all-cause mortality: Systematic review and meta-analysis. BMC Med..

[7] Perera S., Mody S.H., Woodman R.C., Studenski S.A. (2006). Meaningful Change and Responsiveness in Common Physical Performance Measures in Older Adults. J. Am. Geriatr. Soc..

[8] Pahor M., Guralnik J.M., Ambrosius W.T., Blair S., Bonds D.E., Church T.S., Espeland M.A., Fielding R.A., Gill T.M., Groessl E.J. (2014). Effect of structured physical activity on prevention of major mobility disability in older adults: The LIFE study randomized clinical trial. J. Am. Med. Assoc..

[9] Fielding R.A., Rejeski W.J., Blair S., Church T., Espeland M.A., Gill T.M., Guralnik J.M., Hsu F.-C., Katula J., King A.C. (2011). The Lifestyle Interventions and Independence for Elders Study: Design and Methods. J. Gerontol. Ser. A Biol. Sci. Med. Sci..

[10] Layne A.S., Hsu F.-C., Blair S.N., Chen S.-H., Dungan J., Fielding R.A., Glynn N.W., Hajduk A.M., King A.C., Manini T.M. (2017). Predictors of Change in Physical Function in Older Adults in Response to Long-Term, Structured Physical Activity: The LIFE Study. Arch. Phys. Med. Rehabil..

[11] Nagin D.S., Odgers C.L. (2010). Group-Based Trajectory Modeling in Clinical Research. Annu. Rev. Clin. Psychol..

[12] White D.K., Neogi T., Nevitt M.C., Peloquin C.E., Zhu Y., Boudreau R., Cauley J.A., Ferrucci L., Harris T.B., Satterfield S.M. (2012). Trajectories of Gait Speed Predict Mortality in Well-Functioning Older Adults: The Health, Aging and Body Composition Study. J. Gerontol. Ser. A Biol. Sci. Med. Sci..

[13] Laddu D.R., Parimi N., Cauley J.A., Cawthon P.M., Ensrud K.E., Orwoll E., Stefanick M., Langsetmo L. (2018). Osteoporotic Fractures in Men (MrOS) Study Research Group; for the Osteoporotic Fractures in Men (MrOS) Study Research Group The Association Between Trajectories of Physical Activity and All-Cause and Cause-Specific Mortality. J. Gerontol. Ser. A Biol. Sci. Med. Sci..

[14] Han L., Gill T.M., Jones B.L., Allore H. (2015). Cognitive Aging Trajectories and Burdens of Disability, Hospitalization and Nursing Home Admission Among Community-living Older Persons. J. Gerontol. Ser. A Biol. Sci. Med. Sci..

[15] De La Fuente J., Caballero F.F., Sanchez-Niubo A., Panagiotakos D.B., Prina A.M., Arndt H., Haro J.M., Chatterji S., Ayuso-Mateos J.L., Prina M.A. (2018). Determinants of Health Trajectories in England and the United States: An Approach to Identify Different Patterns of Healthy Aging. J. Gerontol. Ser. A Biol. Sci. Med. Sci..

[16] Marsh A.P., Lovato L.C., Glynn N.W., Kennedy K., Castro C., Domanchuk K., McDavitt E., Rodate R., Marsiske M., McGloin J. (2013). Lifestyle interventions and independence for elders study: Recruitment and baseline characteristics. J. Gerontol. Ser. A Biol. Sci. Med. Sci..

[17] Rejeski W.J., Axtell R., Fielding R., Katula J., King A.C., Manini T.M., Marsh A.P., Pahor M., Rego A., Tudor-Locke C. (2013). Promoting physical activity for elders with compromised function: The lifestyle Interventions and Independence for elders (LIFE) study physical activity intervention. Clin. Interv. Aging.

[18] Jones B.L., Nagin D.S., Roeder K. (2001). A SAS Procedure Based on Mixture Models for Estimating Developmental Trajectories. Sociol. Methods Res..

[19] Pahor M., Guralnik J.M., Anton S.D., Ambrosius W.T., Blair S.N., Church T.S., Espeland M.A., Fielding R.A., Gill T.M., Glynn N.W. (2020). Impact and Lessons From the Lifestyle Interventions and Independence for Elders (LIFE) Clinical Trials of Physical Activity to Prevent Mobility Disability. J. Am. Geriatr. Soc..

[20] Resnick B., Boltz M. (2020). The Impact of Psychological Status, Social Well-Being, and Physical Function on Healthcare Utilization. J. Am. Geriatr. Soc..

[21] Riebe D., Franklin B.A., Thompson P.D., Garber C.E., Whitfield G.P., Magal M., Pescatello L.S. (2015). Updating ACSM’s Recommendations for Exercise Preparticipation Health Screening. Med. Sci. Sports Exerc..

[22] Cheng Y., Goodin A.J., Pahor M., Manini T., Brown J.D. (2019). Healthcare Utilization and Physical Functioning in Older Adults in the United States. J. Am. Geriatr. Soc..

[23] Brown J.D., Wang C.Y., Groessl E.J., Pahor M., Manini T. (2020). Three-year post-intervention follow-up comparison of healthcare resource utilization and costs in the Lifestyle Interventions and Independence for Elders (LIFE) Study. J. Gerontol. Ser. A Biol. Sci. Med. Sci..

